# Postoperative Complications following Nodal Dissection and Their Association with Melanoma Recurrence

**DOI:** 10.1155/2013/382138

**Published:** 2013-02-26

**Authors:** Abubakr Ahmed, Gaitri Sadadcharam, Felicity Huisma, Katrina Fogarty, Muhammad Mushtaque, Azher Shafiq, Paul Redmond

**Affiliations:** Department of Academic Surgery, University College Cork (UCC), National University of Ireland (NUI), Cork University Hospital, Cork, Ireland

## Abstract

*Background*. Although postoperative complications are common after lymph node dissection, its association with disease recurrence has not yet been fully investigated. *Methods*. A retrospective review of a prospectively maintained database was conducted, looking at all malignant melanoma patients with sentinel nodes positive disease requiring axillary or inguinal dissection between 2002 and 2011. *Results*. A total of 124 patients required nodal clearance from 317 patients with stage I/II malignant melanoma who had undergone sentinel lymph node biopsy. Of these, 104 patients met the inclusion criteria and were divided into inguinal lymph node dissections (ILND; *n* = 63) or axillary lymph node dissections (ALND; *n* = 41). Immunohistochemical deposits had higher detection rate in ALND (*P* = 0.01). The ILND patients had a higher recurrence rate (84.1% versus 63.4%; *P* = 0.02) and mortality (68.3% versus 48.8%; *P* = 0.05) without a significant difference in complications. In patients whom complications developed, 75% of the ILND group and 71.4% of the ALND group had disease recurrence, but without reaching a statistical value as an independent predictor of melanoma recurrence. *Conclusion*. Complications are common following ILND and ALND; however there is no significant difference in complications rates between the groups with some associations with recurrence without reaching a significant difference.

## 1. Introduction

Malignant melanoma has a unique position in the surgical and oncological fields as it is responsible for 79% of mortalities despite representing less than 5% of cutaneous malignancies [1]. The considerable variation in recurrence and survival rates among melanoma patients has motivated many researchers to look at different variables and risk factors that could predict prognosis. Morton et al. revolutionised the management of malignant melanomas in early 1990s by developing the sentinel lymph node biopsy (SLNB) technique, an important landmark in the management of clinical stage I and II cutaneous melanoma [2]. As a result, the Multicentre Selective Lymphadenectomy Trial (MSLT) was initiated [3]. This trial has shown that the presence of metastases in the sentinel node was the most important prognostic factor and has recommended completion lymph node dissection for patients with a positive SLNB [3].

There are several histopathological features that predict recurrence and survival, including the Breslow's thickness, the presence of ulceration, and the mitotic rate [4]. Age, sex, presence of lymphovascular invasion and SLN status are other recognised risk factors [5]. The impact of the anatomical location of the primary melanoma on prognosis has been a matter of controversy, but some studies have suggested that truncal and head and neck melanomas have a worse prognosis compared to other sites [6–8]. Recent reports have shown that head and neck melanomas tend to present with macrometastases compared with truncal melanomas which are associated with micrometastases [9]. Furthermore, melanomas on the upper limb tend to be thinner compared to other sites, with longer survival rates but without an identifiable reason [10].

Axillary and inguinal lymph node dissections are performed when lymph basin is involved and are associated with significant morbidities including infection, wound dehiscence, bleeding, deep vein thrombosis, and most importantly lymphedema. A better understanding of the preoperative risk factors could help predict which patients are more susceptible to postoperative complications. Furthermore, the development of complications in itself may have an impact on the recurrence and survival rates of patients. Up to date, there is little data on the complication rates comparing different dissection techniques. The only large trail that investigated the impact of morbidity following lymphadenectomies found strong association between lymphedema with groin dissection (26.6%) than that with axillary dissection (9%) [11].

Several trials including the MSLT compared the complications rates between different techniques in the similar lymph node groups. For example, in patients undergoing SLNB, comparisons were made between (1) wider margins with SLNB, (2) wider excision margins alone or (3) completion lymph nodes dissection [12, 13]. In addition, a comparison between immediate and delayed lymph node dissection has also been reported [3]. To our knowledge, no studies to date have examined the risk factors for complications or the role of complications, as being a predictor of melanoma recurrence. This is particularly important considering that the gold slandered treatment is very invasive and associated with high risk of complications and morbidity. 

Thus, the primary objective of this study was to identify preoperative risk factors for postoperative complications. A secondary end point was to investigate the postoperative complications following ALND or ILND as a possible predictor for melanoma recurrence.

## 2. Methods

A prospectively maintained database from 2002 to 2011 was retrospectively reviewed. The presented data is of a single surgeon in a tertiary melanoma referral centre where we receive referrals from all hospitals in the southern provinces in Ireland. A total of 317 patients underwent either an inguinal or axillary SLNB as a staging procedure in Cork University Hospital for stage I/II malignant melanoma (Breslow >1 mm) in this time period. All patients who subsequently went on to either an axillary or inguinal lymph node dissection due to a positive SLN and who had no evidence of systemic metastases were included. 

The analysed data included patient-related variables such as gender, age at diagnosis, and primary melanoma site. The histology of the primary lesion was reviewed for melanoma type, Clark's level, Breslow thickness, presence of ulceration, lymphovascular invasion, and mitotic index. All positive SLNs were reviewed to determine the type of metastases. The lymph node dissection samples were examined for the total number of nodes and the number of positive nodes. Data from patients who developed disease recurrence was examined to determine the site of locoregional recurrence as well as the duration from the time of original diagnosis to recurrence. Finally, postoperative complications, distant metastases and patient overall survival were recorded. 

ALND involved level I and level II clearance. A level III clearance, with preservation of pectoralis minor, was performed if suspicious intraoperative nodes were found. An infrainguinal lymph node dissection was performed as standard, unless there was evidence of suprainguinal lymph node involvement.

The sentinel lymph node samples were sent for routine histopathological examination with hematoxylin and eosin (H&E) staining. H&E negative nodes were further processed with specific immunohistochemical stains for protein S100 and melanoma-related antigen HMB45. Patients with positive nodes by either H&E staining or on immunohistochemistry were scheduled for immediate lymph node dissection. 

### 2.1. Statistical Analysis

The statistical analysis was performed using SPSS 20 (SPSS Inc. Chicago, IL, USA). Descriptive statistics such as rates and percentages were used for categorical data while mean ± standard deviations (SD) were used for continuous data. The categorical variables were tested using *χ* ^2^ test, Fisher's exact test, or the Wilcoxon rank-sum test. Continuous variables were tested using the Student's *t*-test. Standard logistic regression analysis was used to calculate the relative risk as odds ratios (OR with 95% confidence intervals (CI). A *P* value of less than or equal to 0.05 was considered significant.

## 3. Results

Using the inclusion criteria, a total of 124 suitable patients were identified. Twenty patients were excluded either because of missing data with regard to their primary histology or because of follow-up loss. The remaining 104 patients were included in the final analysis. These patients were divided into the ILND group (60.6%; *n* = 63) and the ALND group (39.4%; *n* = 41). The average age profile of patients in the ILND was older compared to the ALND group (57.4 ± 16.70 versus 50 ± 16.06 years; *P* = 0.03). The follow-up period ranged from 6 months to nine years. Males were found to have a higher incidence of upper limb melanomas requiring ALND whereas females had a higher incidence of lower limb melanomas requiring ILND (*P* = 0.08). In our population group lower limb melanomas were noted to have a higher Breslow thickness (5.03 ± 4.49) compared with melanomas of the upper limb (3.8 ± 3.06; *P* = 0.11). The demographic and pathological characteristics of both groups are described in Table 1. The detection of micro- and macrometastases after SLNB was comparable in both groups. However, the ALND group had a higher rate of immunohistochemistry detected metastatic deposits (*P* = 0.01). 

Patients who underwent ILND had a higher mortality rate (68.3% versus 48.8%; *P* = 0.05) compared to the ALND group. This is most likely a reflection of the higher recurrence rate in the ILND group as seen in Table 2 (ILND (84.1%) versus ALND (63.4%); *P* = 0.02). 

It was noted that the ILND group had relatively more complications compared to the ALND group (39.7% versus 34.1% resp.; *P* = 0.57). The development of lymphedema and seroma formation was more common in the ILND group. Wound infection rates were higher in the ALND group (Table 3). 

We then went on to look for correlations between preoperative factors and complications. We found that male patients were more likely to develop complications after ALND compared to females in both univariate and multivariate analysis (*P* = 0.03, 95% CI 0.03–0.96). With regard to patients in the ILND group, Clark's level was a predictor of postoperative complications. Patients with a Clark's level of 4 or more carried a higher risk of developing complications postoperatively (*P* = 0.05, 95% CI 0.96–15.16) (Table 4). Despite the relative high complication rates in both groups, the impact on the development of local or distal metastases was not significant. However, most patients who did develop complications after ILND had a recurrence of their melanoma (*n* = 19/25, 75%). Similarly, 10 out of 14 patients (71.4%) who developed complications after ALND had melanoma recurrence (Table 5).

## 4. Discussion

Lymph node dissections carry significant morbidity with high rates of both acute and chronic complications. These complications negatively impact on the overall function and quality of life of these patients and also pose a significant financial burden to the health system. Surgery, in itself, has been implicated as a potential promoting factor in the propagation of malignant lesions [14, 15] and may also suppress cell-mediated immunity [16]. Taking this into account, both surgery and the complications of surgery can synergistically combine to facilitate the establishment of new metastases and the progression of preexisting micrometastases [17]. For these reasons, we hypothesise that high complication rates following lymph node dissection might have an effect on metastases and could be used as a prognostic indicator for recurrence. 

The anatomical location of the primary lesion has been reported as an independent predictor of a positive SLN and disease-free survival. However, this role is limited when compared to other well-established variables [10]. Our results show an increased incidence of upper limb and truncal melanomas in male patients whereas females had an increased incidence of lower limb melanomas. With regards to Breslow thickness, it was observed that patients with upper limb melanomas had thinner tumours and a better overall survival. These findings are in accordance with previous reports [9, 10]. The anatomical distribution and its relation to gender could be explained by different sun exposure patterns between genders. In addition, upper limb lesions are more likely to be noticed at an earlier stage, and thus treatment can be initiated sooner. This could account for the preponderance of thinner lesions in the upper limbs.

A significant amount of heterogeneity was observed in our data among patients with nodal basin metastases. According to the MSLT, node-positive patients who underwent an immediate lymphadenectomy had a longer disease-free survival with a lower risk of recurrence [18, 19]. The long-term survival was also observed to be extended when a lymphadenectomy was performed for micrometastases compared to being performed in the setting of clinically detectable nodal disease [20]. Unfortunately, we reported a high mortality rate in our patients with no clinically detected nodes who went on to have an immediate lymphadenectomy. This is most likely explained by the disease stage at presentation; 49% of our patients had a Breslow thickness of >3.5 mm, which is the upper limit in most trials as it has been associated with a poor outcome [3]. Nonetheless, we included this category of patients in our study as it is a real-time reflection of our overall practice and to avoid any selection bias. 

It has been reported that morbidity is higher in patients undergoing inguinal lymphadenectomy compared to those undergoing axillary or cervical lymph node dissection [21]. However, in the literature, there is no difference in the complication rates between ALND and ILND [22] which correlated with the findings in this study (39.7% in ILND versus 34.1% in ALND, *P* = 0.57). 

The literature was reviewed with particular emphasis on differences between the dissection groups. In the ILND group there was no significant difference in early postoperative mortality. A trend for developing lymphedema was noted in the deep dissection group [23]. Despite the higher rate of regional recurrence after superficial dissections compared to deep dissections no significant difference was seen in overall survival rates [24]. The published rate of complications following groin dissection is quite variable. Using wound infection rates as an example, our 9.5% infection rate is midway in the published figures which range from 5 to15% [25]. Similarly, our lymphedema rate of 22.2% is comparable to the published range of 21–40% [25, 26].

Variations in surgical technique of ALND have a minimal effect on the development of complications. For example, dissections either above or below the axillary vein do not increase the complication rate [27] with an incidence of long term lymphedema between 1–12% [12, 27]. We report an incidence of 14.6% for both lymphedema and seroma formation in our study. It is worthwhile mentioning that due to the high incidence of seroma formation after axillary surgery, some authors consider it as a consequence of the surgery other than a direct complication [28]. 

Our results did not show a statistical significant correlation between the development of postoperative complications and the risk of recurrence of melanoma. It is important to remember that this data applies to a cohort of patients with relatively very thick melanomas which inheritably carries a higher recurrence rate. However, it is not yet known if this data applies to patients with “thinner” melanomas. 

In conclusion, the overall complication rate is comparable for both groin and axillary dissections. Male patients tend to have more complications after axillary clearance compared to females. In the ILND group, a Clark's level of 4 or more is a predictor for the development of complications. Finally this study shows that patients who developed complications had a higher incidence of melanoma recurrence; however, this did not reach significance and hence may not represent a true association.

## Figures and Tables

**Table 1:** Demographic and pathological characteristics.

Characteristics	Total (*N* = 104)	Inguinal dissection (*N* = 63)	Axillary dissection (*N* = 41)	*P* value
Gender				
Male	55 (52.9%)	29 (46%)	26 (63.4%)	0.083
Female	49 (47.1%)	34 (54%)	15 (36.5%)	
Age				
Mean ± SD	54.5 (±16.98)	57.4 (±16.70)	50 (±16.06)	**0.029**
Median	56	60	51	
Primary site				
Extremity	88 (84.6%)	61 (96.8%)	27 (65.9%)	**<0.001**
Trunk	16 (15.3%)	2 (3.2%)	14 (34.1%)	**<0.001**
Breslow (mm)				
Mean ± SD	4.55 (±4.01)	5.03 (±4.49)	3.8 (±3.06)	0.11
Median	3.5	3.8	3	
1–1.79 mm	17 (16.3%)	10 (15.9%)	7 (17.1%)	0.872
1.80–3.5 mm	36 (34.6%)	19 (30.2%)	17 (41.5%)	0.236
>3.5 mm	51 (49%)	34 (54%)	17 (41.5%)	0.213
Clarke's level				
III or >	26 (25%)	16 (25.4%)	10 (24.4%)	0.908
IV	67 (64.4%)	38 (60.3%)	29 (70.7%)	0.278
V	11 (10.6%)	9 (14.3%)	2 (4.9%)	0.127
Ulceration				
Present	40 (38.5%)	27 (42.9%)	13 (31.7%)	0.235
Absent	35 (51%)	29 (46%)	24 (58.5%)	0.213
Unknown	11 (10.6%)	7 (11.1%)	4 (9.75%)	0.826
Lymph vascular invasion				
Present	18 (17%)	12 (19%)	6 (14.6%)	0.561
Absent	72 (69.2%)	43 (68.3%)	29 (70.7%)	0.789
Unknown	14 (13.5%)	8 (12.7%)	6 (14.6%)	0.777
Melanoma type				
Superficial spreading	51 (49%)	29 (46%)	22 (53.7%)	0.447
Nodular	29 (27.9%)	16(25.4%)	13 (31.7%)	0.483
Lentigo maligna	7 (6.7%)	4 (6.3%)	3 (7.3%)	0.847
Acral lentiginous	13 (12.5%)	11 (17.5%)	2 (4.9%)	**0.05**
Amelanotic	4 (3.8%)	3 (4.8%)	1 (2.4%)	0.547

**Table 2:** Nodal characteristics, recurrence and survival.

Characteristics	Inguinal dissection (*N* = 63)	Axillary dissection (*N* = 41)	*P* value
Sentinel node			
Macrometastasis	18 (28.6%)	13 (31.7%)	0.772
Micrometastasis	44 (69.8%)	22 (53.7%)	0.106
Immunohistochemical	1 (1.6%)	6 (14.4%)	**0.01**
Total nodes (mean ± SD)	11.45 (±4.9)	19.76 (±10.8)	**<0.001**
Median of total nodes	11	18	—
Positive nodes (mean ± SD)	1.93 (±3.25)	2.12 (±3.27)	0.722
Median of positive nodes	1	1	—
% of positive nodes (mean ± SD)	16.84 (±24.56)	14.37 (±24.87)	0.615
Recurrence rate	53 (84.1%)	26 (63.4%)	**0.016**
Mean time to recurrence (months)	28.9 (±24)	34 (±27.7)	0.206
Site of recurrence			
Local or in transit	13 (20.6%)	6 (14.6%)	0.887
Nodal	16 (25.4%)	7 (17.5%)	0.764
Distant	42 (66.7%)	22 (53.7%)	0.567
Mortality	43 (68.3%)	20 (48.8%)	**0.047**

**Table 3:** Complications rate.

Complications	GLND (*N* = 63)	ALND (*N* = 41)	*P* value
Overall	25 (39.7%)	14 (34.1%)	0.569
Lymphedema	14 (22.2%)	6 (14.6%)	0.337
Wound infection	6 (9.5%)	6 (14.6%)	0.425
Seroma	13 (20.6%)	6 (14.6%)	0.439
Haematoma	2 (3.2%)	1 (2.4%)	0.827
Chronic pain	2(3.2%)	0	0.249

**Table 4:** Analysis of risk factors associated with complications.

Risk factors for complications	ILND	*P* value	ALND	*P *value	ILND versus ALNC
Age > 60	16/32 (50%)	0.089	6/14 (42.9%)	0.397	0.655
Male	11 (37.9%)	0.793	12 (46.2%)	**0.033**	0.357
Female	14 (41.2%)	—	2 (13.3%)	—	0.055
Extremity	24 (39.3%)	0.762	8 (29.6%)	0.397	0.382
Trunk	1 (50%)	—	6 (42.8%)	—	0.849
LVI	7 (58.3%)	0.935	3 (50%)	0.954	0.778
Ulceration	13 (48.1%)	0.324	5 (38.5%)	0.228	0.131
Breslow > 3.5	12 (35.3%)	0.441	5 (29.4%)	0.591	0.674
Clark > 4	20 (42.6%)	**0.048**	10 (32.2%)	0.653	0.201
Positive nodes > 30%	4/12 (25%)	0.935	3/7 (42.8%)	0.558	0.783
Melanoma type					
Superficial spreading	11 (37.9%)	0.793	8 (36.4%)	0.747	0.909
Nodular	7 (43.8%)	0.700	4 (30.8%)	0.756	0.474
Lentigo maligna	1 (25%)	0.535	2 (66.7%)	0.217	0.270
Acral lentiginous	4 (36.4%)	0.804	0	0.296	0.305
Amelanotic	2 (66.7%)	0.328	0	0.466	0.248

**Table 5:** The association between complications and recurrence.

Recurrence risk	ILND (63)	*P* value	ALND (41)	*P* value
Overall recurrence	53 (84.1%)	**0.016**	26 (63.4%)	—
Overall complications	25 (39.7%)	0.569	14 (34.1%)	—
Complication with recurrence	19 (30.1%)	0.152	10 (24.3%)	0.433
Lymphedema	11 (17.4%)	0.519	5 (12.2%)	0.273
Wound infection	5 (7.9%)	0.995	4 (9.7%)	0.858
Seroma	11 (17.4%)	0.957	4 (9.7%)	0.858
Haematoma	2 (3.2%)	0.532	19 (2.4%)	0.442
Chronic pain	1 (1.6%)	0.180	0	0.442
